# Assessment of malaria risk in Southeast Asia: a systematic review

**DOI:** 10.1186/s12936-023-04772-3

**Published:** 2023-11-08

**Authors:** Chaitawat Sa-ngamuang, Saranath Lawpoolsri, Myat Su Yin, Thomas Barkowsky, Liwang Cui, Jetsumon Prachumsri, Peter Haddawy

**Affiliations:** 1 Faculty of Information and Communication Technology, Mahidol University, Nakhon Pathom, Thailand; 2 Department of Tropical Hygiene, Faculty of Tropical Medicine, Mahidol University, Bangkok, Thailand; 3 Bremen Spatial Cognition Center (BSCC), University of Bremen, Bremen, Germany; 4 Division of Infectious Diseases and International Medicine, Department of Internal Medicine, Morsani College of Medicine, University of South Florida, Tampa, USA; 5 Mahidol Vivax Research Unit, Faculty of Tropical Medicine, Mahidol University, Bangkok, Thailand

**Keywords:** Malaria, Risk, Low-transmission areas, Southeast Asia

## Abstract

**Background:**

Several countries in Southeast Asia are nearing malaria elimination, yet eradication remains elusive. This is largely due to the challenge of focusing elimination efforts, an area where risk prediction can play an essential supporting role. Despite its importance, there is no standard numerical method to quantify the risk of malaria infection. Thus, there is a need for a consolidated view of existing definitions of risk and factors considered in assessing risk to analyse the merits of risk prediction models. This systematic review examines studies of the risk of malaria in Southeast Asia with regard to their suitability in addressing the challenges of malaria elimination in low transmission areas.

**Methods:**

A search of four electronic databases over 2010–2020 retrieved 1297 articles, of which 25 met the inclusion and exclusion criteria. In each study, examined factors included the definition of the risk and indicators of malaria transmission used, the environmental and climatic factors associated with the risk, the statistical models used, the spatial and temporal granularity, and how the relationship between environment, climate, and risk is quantified.

**Results:**

This review found variation in the definition of risk used, as well as the environmental and climatic factors in the reviewed articles. GLM was widely adopted as the analysis technique relating environmental and climatic factors to malaria risk. Most of the studies were carried out in either a cross-sectional design or case–control studies, and most utilized the odds ratio to report the relationship between exposure to risk and malaria prevalence.

**Conclusions:**

Adopting a standardized definition of malaria risk would help in comparing and sharing results, as would a clear description of the definition and method of collection of the environmental and climatic variables used. Further issues that need to be more fully addressed include detection of asymptomatic cases and considerations of human mobility. Many of the findings of this study are applicable to other low-transmission settings and could serve as a guideline for further studies of malaria in other regions.

## Background

Malaria remains the most serious life-threatening vector-borne disease. Approximately 240 million cases of malaria infection and 620,000 deaths were reported worldwide in 2020. Despite the high global incidence, some regions have made significant progress. Several countries in Southeast Asia, such as Thailand, Malaysia, and Indonesia, are nearing malaria elimination [1, 2]. Yet, many challenges exist in achieving the last mile of malaria elimination. In particular, it requires targeted elimination efforts, where risk prediction can play a supporting role.

Tracking progress through surveillance is essential to target elimination efforts [3], but effective surveillance faces challenges in near-elimination areas. Asymptomatic cases typically represent a small percentage of all malaria cases (less than 5%) [1], and the importance of detecting them increases in areas nearing elimination. Detection of asymptomatic cases requires active surveillance, which entails a high input of effort and costs. Furthermore, the high spatial and temporal heterogeneity of malaria cases in low-transmission settings can result in small areas of relatively high transmission. Both these factors mean that surveillance must be highly targeted. In addition, the importation of malaria cases from high-incidence areas of neighboring countries poses a further challenge. Accurate spatiotemporal risk estimates are essential in identifying transmission hotspots and potential importation routes, which are needed to inform control agencies to focus surveillance and control efforts.

Despite its importance, there is no standard numerical method to quantify the risk of malaria infection, and no acceptable risk level is advised [4]. As a result, each study of risk selects or establishes its own definition of the risk of malaria infection and designs a quantitative method to measure it, leading to incomparable results. Thus there is a need for a consolidated view of existing definitions of risk and factors/predictors considered in assessing risk to analyse the merits of risk prediction models, particularly in low transmission areas.

**Fig. 1:**
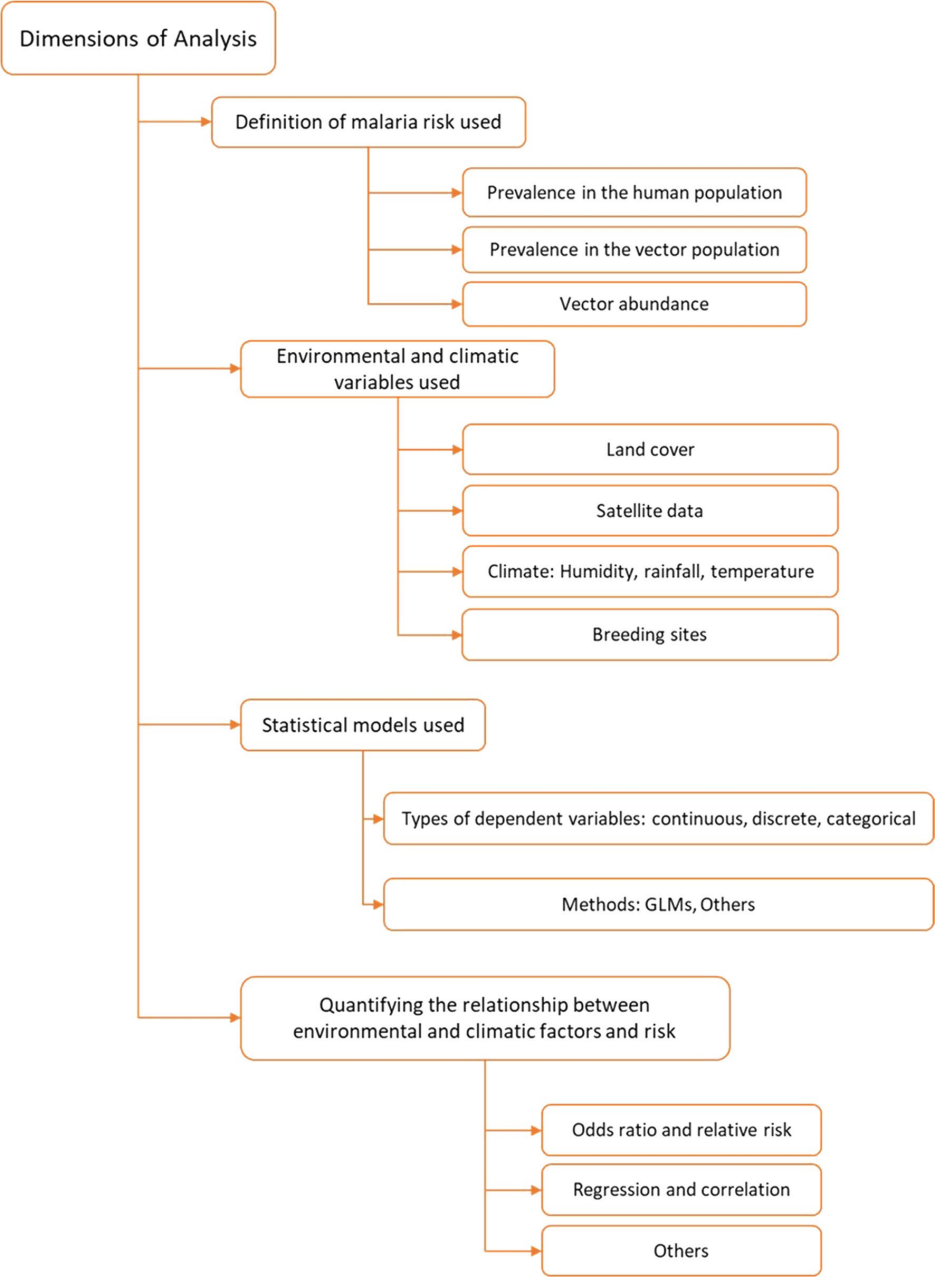
An overview of dimensions of analysis in each paper

The risk of malaria infection in a region is typically defined in terms of prevalence (proportion of malaria cases) or entomological inoculation rate (the infective biting per time unit). Due to the labour-intensive nature of collecting such data, risk models commonly use environmental and climatic factors to infer the risk because malaria transmission is highly dependent on them [1]. This systematic review thus focuses on such models of risk, examining studies of risk in Southeast Asia with regard to their suitability in addressing the challenges of malaria elimination in low transmission areas. Factors examined include the definition of the risk of malaria infection used in each study, the spatial and temporal granularity, the environmental and climatic factors associated with the risk, the analysis techniques used to infer risk, and the generalizability of the approach. Figure 1 provides an overview of the dimensions analysed in each paper included in this review. This systematic review aims to serve as a guideline for malaria epidemiology studies in low-transmission settings.

## Methods

### Inclusion criteria

- The search terms are contained in the title, abstract, or keywords
- Studies focus on utilizing environment and weather as predictors of risk
- Studies are conducted in Southeast Asia region [5–7] (Thailand, Myanmar, Vietnam, Laos, Cambodia, Philippines, Malaysia, Indonesia, Singapore, Timor-Leste, and Brunei)
- Studies are peer-reviewed articles or proceedings papers
- Studies are written in English.

### Exclusion criteria

- Studies have irrelevant titles or abstracts. For example, this includes studies that mainly explore other vector-borne diseases or focus on drug experimentation or the evaluation of treatment schemes
- Full papers are not accessible
- Studies examine other risk factors, such as behavioural, serological, or genetic material factors, without mentioning environmental factors
- Studies are literature reviews, systematic reviews, or protocols

### Search terms

The search terms were defined to select studies involving malaria, environmental and climatic factors, risk, and the Southeast Asia region [5–7]. The search used was: malaria AND (“risk factors” OR “risk areas” OR “risk”) AND (“environment” OR “environmental” OR “environmental factors” OR “landcover” OR “land cover” OR “land-cover” OR “land covers” OR “land cover types” OR “land use” OR “land-use” OR “landscape”) AND (“Southeast Asia” OR Thailand OR Myanmar OR Vietnam OR Laos OR Cambodia OR Philippines OR Malaysia OR Indonesia OR Singapore OR Timor-Leste OR Brunei). The duration of publication was limited to 10 years (2010–2020). Four electronic databases were searched: PubMed, EMBASE (Medline), Web of Science, and Google Scholar.

### Appraisal of the articles

The estimation of the risk of malaria based on environmental and climatic factors requires a study to select (i) a definition of risk of malaria infection, (ii) the environmental and climatic variables to use, (iii) statistical models, and (iv) quantification approach to explore the relationship between environmental and climatic factors, and risk. Each of the studies was examined according to these criteria.

## Results

### Search and selection strategy

**Fig. 2:**
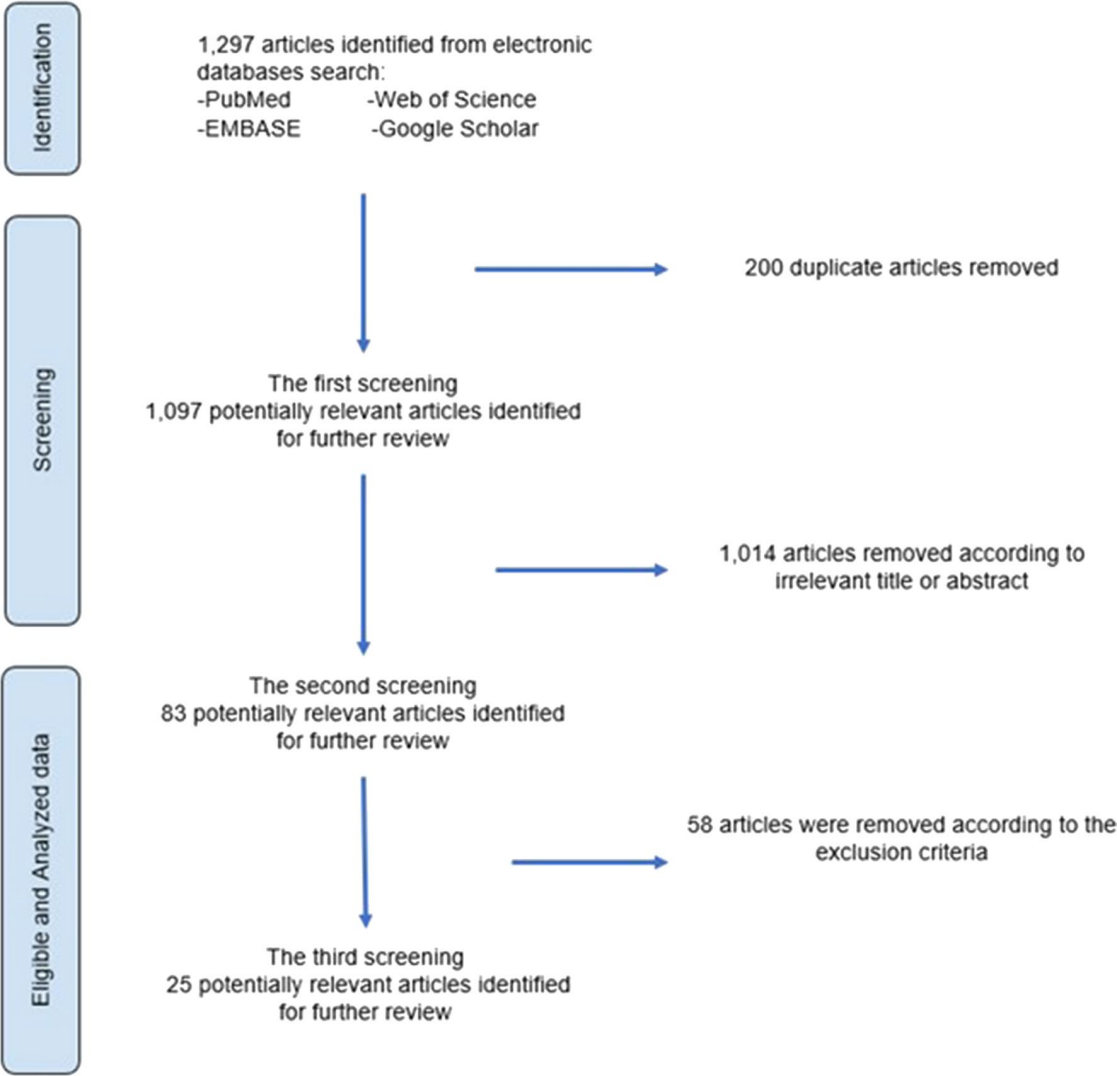
Search and selection process

Figure 2 shows an overview of the search for articles. Use of the search terms and inclusion criteria resulted in 1297 articles being retrieved. The EndNote software (version 10) [8] was used to remove ineligible articles based on the exclusion criteria. Examination identified 200 duplicate articles, which were excluded accordingly. This left 1097 articles for further selection based on the titles and the abstracts. A total of 1014 articles were removed because they had irrelevant titles or irrelevant descriptions in the abstracts. Of the 83 articles left for further selection, 58 were excluded: four were literature reviews, systematic reviews, or research protocols, four were conducted outside Southeast Asia, 21 did not have the full manuscripts accessible, 27 were descriptive studies of other factors, such as serological factors, and two had different titles when the manuscripts were accessed. After the third screening, 25 articles were left for analysis.

### Definition of risk and indicators of malaria transmission

Among the 25 articles selected, nine studies were conducted in Malaysia, four in Thailand, four in China along the border with Myanmar, three in Cambodia, and two each in Indonesia, Lao PDR, and Vietnam (Table 1). All the studies examined directly used an indicator of malaria transmission in a region as their definition of risk. The studies used three indicators to measure the degree of malaria transmission: (1) the prevalence of malaria infection in the human population, (2) the prevalence of the parasite in the vector population, and (3) measures of vector abundance as proxy measures. The articles corresponding to each approach are discussed in turn. A summary of the articles is provided in Table 2.

**Table 1:** Articles categorized by definition of risk

Categories	References	Source of data	Parasite detection method	Period of data collection	Time resolution	Spatial resolution	Study site(s)
Prevalence	Nixon et al. [9]	CSS	Microscopy	1 year	Yearly reports	Household	Indonesia
	Fornace et al. [13]	CSS	Microscopy and PCR	1 year	Yearly reports	Village	Malaysia/Philippines
	Ninphanomchai et al. [20]	PCD: malaria cases	Microscopy	10 years	Monthly reports	District	Thailand
	Sluydts et al. [14]	CSS	PCR	1 year	Yearly reports	Village	Cambodia
	Lawpoolsri et al. [15]	PCD: malaria cases	Microscopy	7 years	Monthly reports	Village	Thailand (TMB)
	Zhao et al. [16]	PCD: malaria cases	Microscopy	5 years	Monthly reports	Village	China (CMB)
	Fornace et al. [10]	CSS	PCR	1 year	Yearly reports	Household	Malaysia
	Jeffree et al. [11]	CSS	Microscopy	1 year	Yearly reports	Household	Malaysia
	Okami and Kohtake [21]	PCD: malaria cases	NA	3 years	Monthly reports	District	Cambodia
	Kaewpitoon et al. [24]	PCD: malaria cases	NA	5 years	Yearly reports	Province	Thailand (TMB)
	Sato et al. [17]	PCD: malaria cases	NA	2 years	Yearly reports	Village	Malaysia
	Hasyim et al. [18]	PCD: malaria cases	RDT and microscopy	1 year	Yearly reports	Village	Indonesia
	Mercado et al. [12]	CSS	Microscopy	3 years	Monthly reports	Household	Thailand (TMB)
	Wangdi et al. [22]	PCD: malaria cases	NA	6 years	Yearly reports	District	Vietnam
	Fornace et al. [19]	PCD: malaria cases	Microscopy	4 years	Yearly reports	Village	Malaysia
	Yang et al. [23]	PCD: malaria cases	NA	1 year	Yearly reports	District	China (CMB)
	Xu et al. [25]	PCD: malaria cases	Microscopy	1 year	Yearly reports	Village	China (CMB)
	Inthavong et al. [26]	PCD: malaria cases	Microscopy	1 month	Monthly reports	Village	Lao PDR
	Grigg et al. [27]	PCD: malaria cases	Microscopy and PCR	2 years	Yearly reports	Village	Malaysia
Prevalence of infection in vector population	Durnez et al. [28]	HLC	ELISA of mosquitoes from HLC	1 year	Biting rate by seasons	Village	Cambodia
	Van Bortel et al. [29]	HLC	ELISA of mosquitoes from HLC	Multiple-year cross-sectional	Biting rate by years	Village	Vietnam
Vector abundance or others as a proxy measure	Fornace et al. [30]	HLC	NA	1 year	Biting rate by months	Village	Malaysia
	Ahmad et al. [31]	Larval collection	NA	1 year	Yearly reports	Village	Malaysia
	Zhang et al. [32]	CDC light-traps	PCR	1 year	Monthly reports	Village	China (CMB)
	Tangena et al. [33]	Human-baited double-net trap	NA	2 years	Seasonal reports	Village	Lao PDR

CSS, cross-sectional survey; PCD, passive case detection; CMB, China-Myanmar border; TMB, Thailand-Myanmar border

**Table 2:** Summary of the definition of risk

Definition of risk	Number of studies	References
Risk of infection in the human population	19	[9–27]
Risk of infection in the vector population	2	[28, 29]
Vector abundance or others as a proxy measure	4	[30–33]

#### The prevalence of infection in the human population

The prevalence of infection in the human population is typically expressed as the percentage of the sampled population infected, commonly detected through microscopy and malaria rapid diagnostic test (RDT). A variety of spatial and temporal granularities were used in measuring prevalence. In terms of spatial granularity, four articles reported the prevalence among households [9–12], seven reported the prevalence among villages [13–19], four reported the prevalence among districts [20–23], and one reported the prevalence among provinces [24]. Three studies [25–27] reported the risk in terms of the number of cases at the village (hamlet) level without baseline population adjustment. The measures of the risk of infection also varied according to temporal granularity. Thirteen studies used yearly reports [9–11, 13, 14, 17–19, 22–25, 27], and six studies used monthly reports [12, 15, 16, 20, 21, 26]. There was no particular association between the spatial and temporal granularities.

#### The prevalence of infection in the vector population

The entomological inoculation rate (EIR) is computed by the number of mosquitoes captured by the human landing catch approach per unit of time, such as per night and the distribution of the malaria parasite in the captured mosquitoes. Only two studies [28, 29] used human landing catch and extracted DNA from the captured mosquitoes to estimate the EIR. Both studies collected the EIR at the village level. The study by Durnez et al*.* [28] reported the EIR over 1 year, while the study by Van Bortel et al*.* [29] reported it monthly. Both studies apply enzyme-linked immunosorbent assay (ELISA) to detect *Plasmodium* parasites in the captured mosquitoes.

#### Vector abundance

Studies in this category conducted entomological surveys, such as the collection of larva near households or at the fringe of the forests or the collection of mosquitoes using standard CDC light traps, human landing catch, or cow-baited traps without detecting the parasite. There were five articles in this group, and they all reported their indicators among villages. Fornace et al*.* [30] used human landing catch to collect the biting rate per night over a period of 1 year. Ahmad et al*.* [31] presented the risk with the number of larvae near households collected over 1 year. Zhang et al*.* [32] and Tangena et al*.* [33] measured the abundance of mosquitoes using light traps and human-baited double net traps, respectively.

### Environmental and climatic variables

In terms of environmental factors, 15 articles used land cover types such as types of plantations or crops [16, 17, 24, 25, 27, 33], hilly or flat areas [13, 16, 18, 25, 28], households or forest areas [28, 29, 33], distance to forest or river, and the coverage of forest [10, 12, 15, 16, 19, 32]. Eight collected the characteristics using field observations or existing data such as land cover maps and surveys [17–19, 24, 25, 27–29], while seven articles processed data from satellite images [10, 12, 13, 15, 16, 32, 33]. Three articles used other variables to characterize the environment. Yang et al*.* [23] used rice yield per square kilometre from field observation. Fornace et al*.* [30] used enhanced vegetation Index (EVI), while Okami and Kohtake [21] used normalized difference vegetation index (NDVI), normalized difference water index (NDWI), and topographic wetness index (TWI). The number of reviewed articles grouped by environmental factors is summarized in Table 3.

**Table 3:** Summary of environmental factors

Environmental factors	Number of studies	References
Types of plantations or crops	6	[16, 17, 24, 25, 27, 33]
Hilly or flat areas	5	[13, 16, 18, 25, 28]
Households or forest areas	3	[28, 29, 33]
Distance to forest or river, and the coverage of forest	6	[10, 12, 15, 16, 19, 32]
Others	5	
Rice yield per square kilometer (RYPSK)	1	[23]
Enhanced Vegetation Index (EVI)	1	[30]
Normalized difference vegetation index (NDVI)	1	[21]
Normalized difference water index (NDWI)	1	[21]
Topographic wetness index (TWI)	1	[21]

In terms of climatic factors, three studies investigated only the effect of the climatic factors from field observations or the reports from weather stations without using environmental factors [20, 22]. The other six studies investigated both climatic and environmental factors. The climatic factors included humidity [12, 20, 24], rainfall [12, 18, 20, 23, 24], temperature [12, 20–24], and seasons (wet and dry) [33]. Of all the studies that investigated the effects of climatic factors, two studies used monthly-aggregated data [12, 20], four studies used annually-aggregated data [18, 21, 23, 24], and one study used seasonally-aggregated data [22]. The summarized number of reviewed articles grouped by climatic factors is provided in Table 4.

**Table 4:** Summary of climatic factors

Climatic factors	Number of studies	References
Humidity indices	3	
Monthly-aggregated humidity	2	[12, 20]
Annually-aggregated humidity	1	[24]
Rainfall indices	5	
Monthly-aggregated rainfall	2	[12, 20]
Annually-aggregated rainfall	3	[18, 23, 24]
Temperature indices	6	
Monthly-aggregated temperature	2	[12, 20]
Seasonally-aggregated temperature	1	[22]
Annually-aggregated temperature	3	[21, 23, 24]

Five studies did not use the characteristics of environmental and climatic factors discussed above. Four mentioned mosquito breeding sites near households, such as stagnant water sources or livestock near households [9, 11, 26, 31], and all of the studies collected the data using field observations. One study explored the locations of clusters of infected people along different parts of a river [14].

### Statistical models

This section describes statistical analysis techniques used in the studies to analyse and quantify the relationship between environmental and climatic variables and malaria risk. The analyses can be categorized into three main groups based on the characteristics of the dependent variable (malaria risk). Some studies estimate the prevalence in the population, represented as a continuous or discrete dependent variable. Others estimate the individual risk, represented as dichotomous malaria outcome dependent variable. Thirteen articles adopted techniques to study population-level continuous dependent variables. Examples of continuous dependent variables include risk score generated by a linear combination [16] and the aggregated incidence or prevalence of malaria-infected cases [15, 17, 18, 23]. The techniques include multiple linear regression [24], generalized linear regression [21, 26, 33], generalized linear mixture models [15, 17], generalized linear mixed models with a negative binomial distribution [19], geographically weighted regression (GWR) [18, 23], regression trees (CART) [28], multi-criteria decision analysis (MCDA) [16], Bayesian hierarchical models [10], and Bayesian models with Integrated Nested Laplace Approximation [30]. Four articles applied techniques to investigate population-level discrete dependent variables, such as the integer number of malaria cases in different villages or areas. The models used were negative binomial regression [29], zero-inflated Poisson (ZIP) regression [22], Poisson regression [20], and Pearson's correlation [12]. Finally, five articles estimated the individual risk, represented as dichotomous malaria outcome dependent variable. The techniques included in the studies are logistic regression [11, 13, 27], hierarchical logistic regression [9], and matched univariate and multivariate logistic regression [25]. In addition, three studies performed only descriptive analysis of the abundance of mosquitoes [31, 32] and *Plasmodium* parasites [14].

Aside from the dependent variable, the reviewed articles can be categorized based on statistical methods. Seventeen articles used generalized linear models (GLMs), while eight applied other techniques. A summary of the reviewed articles grouped by the statistical models is provided in Table 5.

**Table 5:** Summary of statistical models

Statistical model	Number of studies	References
Generalized linear model (GLM)	17	
Multiple linear regression	1	[24]
Generalized linear regression model	3	[21, 26, 33]
Generalized linear mixture model	2	[15, 17]
General linearized mixed models with a negative binomial distribution	1	[19]
Geographically weighted regression (GWR)	2	[18, 23]
Negative binomial regression	1	[29]
Zero-inflated Poisson (ZIP) regression	1	[22]
Poisson regression	1	[20]
Logistic regression	3	[11, 13, 27]
Hierarchical logistic regression models	1	[9]
Matched univariate and multivariate logistic regression analyses	1	[25]
Other techniques	8	
Regression tree model (CART)	1	[28]
Pearson's correlation	1	[12]
Multi-criteria decision analysis (MCDA)	1	[16]
Bayesian model	2	[10, 30]
Descriptive analysis (surveillance of the abundance of mosquitoes and Plasmodium parasites)	3	[14, 31, 32]

### Quantifying the relationship between environmental and climatic factors and risk

In the previous section, the main components to quantify the relationship between the characteristics of environment and climate and malaria infection were explored. Here the focus is on the approaches that the studies used to report their results. There are three groups: odds ratio or relative risk (RR), regression/correlation, and other methods. The reviewed articles grouped by the quantification approaches are summarized in Table 6, while the summarized characteristics of the reviewed articles are provided in Table 7.

**Table 6:** Summary of the approaches used to quantify the relationship between environmental and climatic factors and risk

Approaches	Number of studies	References
Odds ratio and a relative risk	10	[9, 11, 13, 15, 19, 22, 25–27, 33]
Correlation, regression, or other coefficients	8	[12, 16, 18, 20, 21, 23, 24, 30]
Others	7	
The report of malaria prevalence	3	[10, 14, 17]
The distribution of mosquitoes	2	[31, 32]
The relative importance index (RI)	1	[28]
The mean density of biting rate	1	[29]

**Table 7:** Variables, data collection methods, analysis techniques, and quantification approaches used in the reviewed articles

References	Environmental and climatic variables	Data collection for the Environmental and climatic variables	Dependent variables	Analysis techniques	Quantification
Nixon et al. [9]	1. Location of households	Field observation	The distance between households and larval habitats	Hierarchical logistic regression models	Odds Ratio or Relative Risk
	2. Location of larval habitats				
Fornace et al. [13]	1. Elevation	Satellite images	Malaria reported cases	Multivariate logistic regression	Odds Ratio or Relative Risk
	2. Distance from house to forest				
Ahmad et al. [31]	1. Location of households	Field observation	The distance between households and larval habitats	The abundance of larva	Others: The percentage of the vectors
	2. Location of larval habitats				
Durnez et al. [28]	The characteristics of land covers in capture locations (forest plot, village)	Field observation	The density of mosquitoes (e.g. man biting rate (MBR))	Non-parametric classification and regression tree models	The relative importance (RI) score of discriminants that affect the mosquitoes' abundance
Ninphanomchai et al. [20]	Monthly meteorological data (rainfall, temperature, and humidity)	Field observation	Malaria reported cases	Poisson regression	Correlation, regression, or other coefficients
Xu et al. [25]	The characteristics of land covers around households (Hilly zone, Larval habitats within 100 m, Vegetation nearby)	Field observation	Malaria reported cases	Matched univariate and multivariate logistic regression analyses	Odds Ratio or Relative Risk
Sluydts et al. [14]	Location of malaria infection cases	Field observation	Malaria reported cases	Spatial clusters of malaria cases	Other: Prevalence of malaria infection from different villages
Lawpoolsri et al. [15]	Light forest coverage	Satellite images	Malaria reported cases	Multivariate generalized linear mixed models	Odds Ratio or Relative Risk
Zhao et al. [16]	1. Forest cover	Satellite images	Risk score of Multi-Criteria Decision Analysis (weighted linear combination)	Multi-criteria decision analysis	Correlation, regression, or other coefficients
	2. Crop land				
	3. Water body				
	4. Elevation				
	5. Urbanization				
	6. Distance to road				
	7. Distance to health facilities				
Fornace et al. [10]	Coverage of forest	Satellite images	Malaria reported cases	Bayesian hierarchical model	Prevalence of malaria
Jeffree et al. [11]	The presence of breed sites (stagnant water)	Field observation	Malaria reported cases	Multiple logistic regression	Odds Ratio or Relative Risk
Okami and Kohtake [21]	1. NDVI	Satellite images	Malaria reported cases	Generalized linear regression model	Correlation, regression, or other coefficients
	2. NDWI			Inverse Distance Weight (for interpolation)	
	3. TWI				
	4. annual average temperature				
Inthavong et al. [26]	The presence of breed sites (cattle near household)	Field observation	Malaria reported cases	Generalized linear regression model	Odds Ratio or Relative Risk
Grigg et al. [27]	The characteristics of land covers (tall grass, young forest, rice paddy field)	Field observation	Malaria reported cases	Logistic regression models	Odds Ratio or Relative Risk
Kaewpitoon et al. [24]	1. Land used (agriculture areas, number of houses, water reservoirs, forest areas)	Field observation	Malaria reported cases	Multiple regression	Correlation, regression, or other coefficients
	2. Anopheles adult density in villages with reported cases				
	3. Average annual rainfall, average annual temperature, and annual relative humidity				
Van Bortel et al. [29]	The characteristics of land covers where the mosquitoes were collected (forest, village, and a route between the forest and village)	Field observation	The density of mosquitoes	Negative binomial regression	The mean density of biting rate per night between of the village and forest areas
Zhang et al. [32]	Land covers: types of forest in villages	Satellite images	The density and diversity of mosquitoes	The abundance of mosquitoes and the indicator of species diversity	The diversity indices of mosquitos (Simpson’s diversity index and Shannon–Wiener’s index)
Sato et al. [17]	1. Wetland	Field observation	Malaria reported cases	Generalized linear mixture model	Prevalence of malaria infection using different land cover types
	2. Monoculture palm oil plantation				
	3. Mosaic oil palm plantation				
	4. Monoculture rubber plantation				
	5. Dense forest				
	6. Degraded forest				
	7. Bush, cropland mosaic				
Hasyim et al. [18]	1. Altitude	Field observation	Malaria reported cases	1. Ordinary least squares	Correlation, regression, or other coefficients
	2. Distance from river			2. Geographically weighted regression	
	3. Distance from lake and pond				
	4. Distance from forests				
	5. Annual rainfall				
Mercado et al. [12]	1. Land covers: forest coverage	Satellite images	Malaria reported cases	Pearson's correlation analysis	Correlation, regression, or other coefficients
	2. Meteorological data: aggregate monthly temp, rainfall, humidity				
Wangdi et al. [22]	Meteorological data (Maximum temperature without lag time)	Field observation	Malaria reported cases	Zero-inflated Poisson regression	Odds Ratio or Relative Risk
Fornace et al. [19]	The coverage of forest	Field observation	Malaria reported cases	General linearized mixed models with a negative binomial distribution	Odds Ratio or Relative Risk
Tangena et al. [33]	1. Immature rubber plants	Satellite images	The density of mosquitoes	Generalized estimating equations	Odds Ratio or Relative Risk
	2. Mature rubber plants				
	3. Villages				
	4. Secondary forests				
	5. Season (wet and dry seasons)				
Fornace et al. [30]	1. EVI	Satellite images	The density of mosquitoes	Bayesian model with Integrated Nested Laplace Approximation	Correlation, regression, or other coefficients
	2. Distance to the forest				
Yang et al. [23]	1. Annual average temperature	Field observation	Malaria reported cases	Geographically weighted regression	Correlation, regression, or other coefficients
	2. Annual cumulative rainfall				
	3. Rice yield per square kilometer				

#### Odds ratio and relative risk

Odds ratio (OR) and relative risk (RR) are widely used (approximately 40%) in earlier studies [9, 11, 13, 15, 19, 22, 25–27, 33]. In an epidemiological setting, both indicators measure the association between exposure and an outcome. In this review, the exposure to malaria risk is an individual staying in presumably high-risk areas, and the outcome is that an individual develops malaria infection. The relative risk is defined as the ratio between the proportion of the population infected among those exposed to risk and the proportion of the population infected among those not exposed to risk. The odds ratio (OR) is considered an approximation of RR when the outcomes of interest are rare [34]. A RR (or OR) of 1.0 means no difference in risk (or odds) of infection between groups of exposed and non-exposed individuals. An RR (or OR) of more than 1.0 indicates an increase in risk (or odds) among exposed individuals and vice versa.

Three studies quantified the relationship between the number of identified malaria-infected people and the presence of mosquito larval habitats near households, such as stagnant ponds created by rain or running streams in forests [9, 11, 26]. Nixon et al*.* [9] reported a reduction in the risk of infection for households located farther than 1.6 km from larval habitat areas of *Anopheles sundaicus* in Indonesia, expressed as an odds ratio of 0.21 [95% confidence interval (CI): 0.14–0.32]. The presence of stagnant ponds, a larval habitat of *Anopheles balabacensis,* resulted in an odds ratio of identified malaria cases of 7.3 (95% CI 1.2–43.5) in a study in Malaysia [11], while the presence of cattle stalls, a larval habitat areas of *Anopheles dirus,* resulted in an odds ratio of 1.78 (95% CI 0.85–3.74) in a study in Lao PDR [26]. All three studies reported that larval habitats found within a distance of 1.6 km from a household increases the odds of malaria-infected individuals compared to households located outside the range.

Five studies quantified the relationship between the number of identified malaria-infected people and the observed environment surrounding households, including the elevation and the coverage of different land cover types such as agricultural vegetation, forest, and villages. Two studies conducted in Malaysia showed that the high rate of deforestation over the past 5 years resulted in an odds ratio of malaria-infected individuals in villages of 2.22 (95% CI 1.53–2.93) [19]. Consistent with the result of another study by Grigg et al*.* [27], the presence of long grass around households, which is considered to be evidence of deforestation, resulted in an odds ratio of malaria-infected individuals of 2.85 (95% CI 1.25–3.46) in Malaysia. Meanwhile, two studies conducted in the Philippines and along the China-Myanmar border investigated malaria transmission by *An. balabacensis*, *An. dirus*, and *Anopheles minimus*. These two studies did not report the effect of deforestation but emphasized the impact of forest coverage and the elevated areas around the households. In the Philippines, Fornace et al*.* [13] reported that households surrounded by more than 30% of forested area within 1 km resulted in an OR of 2.4 (95% CI 1.29–4.46) compared to households surrounded by less than 30% of forested area. The study along the China-Myanmar border reveals that individuals residing in foot-hill and moderate-hill households in Myanmar have an OR of malaria infection of 5.45 (95% CI 2.52–11.8) and 42.82 (95% CI 5.13–315.75) compared to people who possess households located in upper land or mountainous areas [25].

Another study conducted in Lao PDR broadly investigated the distribution of *Anopheles* mosquitoes. The study reported that village areas have an OR of 1.95 (95% CI 1.60–2.39) in the rainy season and 2.76 (95% CI 2.20–3.48) in the dry season of capturing *Anopheles* as compared to secondary forests, which contradicts the other studies. On the other hand, capturing *Anopheles* mosquitoes in a rubber plantation resulted in an OR of 0.46 (95% CI 0.35–0.61) in the rainy season and 0.55 (95% CI 0.40–0.76) in the dry season, as compared to the secondary forest [33]. The author discussed the possibility that the outcome could result from the low capture rate of the *Anopheles* mosquitoes, which is considered a common issue in low-transmission areas [35, 36].

In addition to the effect of the different land cover types, two studies investigated the role of weather in malaria transmission. Lawpoolsri et al*.* [15] reported an OR of malaria infections of 1.05 (95% CI 1.02–1.09) for *Plasmodium vivax* and 1.27 (95% CI 1.23–1.31) for *Plasmodium falciparum* as the mean minimum temperature increases by 1 °C at the Thai-Myanmar border. In Vietnam, Wangdi et al*.* [22] reported that an increment in maximum temperature by 1 °C increased the infection risk of *P. falciparum* by 3.9% (95% CI 3.5–4.3%) and of *P. vivax* by 1.6% (95% CI 0.9–2.0%) [22].

#### Regression and correlation

Two approaches have been mainly used to produce the quantifiers, the regression approach and others. The results are usually shown as weights or coefficients in models. There were eight studies in this category [12, 16, 18, 20, 21, 23, 24, 30].

Five studies applied a group of regression approaches: geographically weighted regression (GWR), Poisson regression, generalized linear regression, and multivariate regression. Two studies adopted the GWR quantifying the relationship between environmental/climatic factors and malaria infections. One study in Indonesia reported significant coefficients of altitude, distance from forests, and rainfall [18]. Another study on the China-Myanmar border quantified the effect of the annual average temperature, annual cumulative rainfall, and rice yield per square kilometer on malaria infections [23]. A study using the Poisson regression reported the significant effect of the maximum/minimum/mean temperature, rainfall, and humidity on malaria infections [20]. Okami and Kohtake adopted a generalized linear regression model to quantify the relationship between the normalized difference vegetation index (NDVI), normalized difference water index (NDWI), topographic wetness index (TWI), annual average temperature, and malaria reports [21]. Kaewpitoon et al*.* [24] applied multivariate regression to quantify the relationship and found a significant association between malaria infections and the forest areas and an average annual relative humidity.

In addition to the regression approaches, three studies applied MCDA and Pearson's correlation analysis to quantify the relationship between environment/climate and malaria infections, while the Bayesian model with Integrated Nested Laplace Approximation to quantify the relationship between environments/weather and the distribution of mosquitoes. The MDCA quantifies the effect of six environmental factors consisting of forest coverage, cropland coverage, distance to a water body, elevation, distance to urbanized areas, and distance to the road [16]. Pearson's correlation was adopted by Mercado et al*.* [12], who identified four significant environmental and climatic factors associated with the risk of malaria infections, including forest coverage, median temperature with a lag time of 1- and 2-month, average temperature with a lag time of 1- and 2-month, and average humidity with the lag time of 2- and 3-month. Fornace et al. [30] adopted the Bayesian model with Integrated Nested Laplace Approximation and found the significant factors consisting of EVI and distance to the forest (100 m) from a village and the distribution of captured mosquitoes (*An. balabacensis*).

#### Other methods

Seven studies included in this review used other quantifiers, including the malaria prevalence, the distribution of mosquitoes, the relative importance index, and the mean biting rate. Fornace et al*.* [10] reported the prevalence of malaria infections within different parts of a village. Sato et al. [17] reported the prevalence of malaria infections found in different land use types, such as palm oil plantations or rubber plantations. Similarly, Sluydts et al*.* [14] reported the prevalence of malaria infections in several villages without statistical analysis. Two studies quantified the number of disease-carrier mosquitoes found in nearby households. Ahmed et al*.* [31] reported the distribution of mosquitoes, while Zhang et al*.* [32] explored the diversity of the mosquitoes between villages and forest areas using the diversity indices of mosquitoes (Simpson’s diversity index and Shannon–Wiener’s index). Durnez et al. [28] adopt the relative importance index score of discriminants to rank the importance between forests and villages that affect mosquito distribution. Van Bortel et al*.* [29] observed the distribution of mosquitoes using the mean biting rate per night.

## Discussion

### Definition of risk

The World Health Organization (WHO) defines malaria risk as the malaria infection rate in a human population [37], which was used in 70% of the reviewed studies. Estimating the malaria risk based on the infection rate captures the disease burden [4, 37]. The reviewed studies obtained the malaria occurrence in humans based on the number of infections from malaria clinics in communities [15, 16, 18, 27], the regional public health offices [12, 17, 19–25], the door-to-door active case detection and screening [9, 11, 13, 14, 26, 30], and national disease registration systems [38–40]. However, the reports of malaria infection from the national disease registration systems may be incomplete or delayed, depending on the strength of the surveillance system in different countries [41].

Approximately 30% of the reviewed studies estimated the risk of malaria from the rate of malaria infection in combination with entomological determinants of malaria, such as estimates of the vector abundance and the prevalence of the *Plasmodium* parasite in *Anopheles* mosquitoes. The diversity of *Anopheles* mosquitoes is very high, and only a subset of the Genus transmits malaria [42, 43]. Thus, it is important to take into account the variation in main malaria vectors within the region (e.g., *An. minimus* and *Anopheles maculatus* in Thailand [35] vs. *Anopheles leucosphyrus* in Malaysia [19]). To provide a more accurate assessment of malaria risk, the vector abundance can be supplemented with an estimate of the distribution of *Plasmodium* parasites in mosquitoes [44], as represented by EIR, which measures the intensity of malaria transmission [45, 46]. Although EIR is informative, an extremely low number of mosquitos carrying malaria parasites in low-transmission areas often hinders the acquisition of EIR. Studies conducted in low transmission areas reported that only approximately 1% of captured mosquitoes had *Plasmodium* parasites [35, 36, 47]. Hence, it is not surprising that only 2% of the studies included in this review reported EIR as an indicator of malaria risk.

In low-transmission settings, a significant contributor to malaria transmission can be the importation of the parasite from high-transmission areas due to human mobility [15, 48, 49]. There are two basic mechanisms of importation. The importation can be caused by infected individuals living in high-transmission areas visiting low-transmission areas or by individuals living in low-transmission areas visiting and becoming infected in a high-transmission area and then bringing the infection back with them when they return home. To quantify the risk of importation, a definition of malaria risk in the high transmission area is needed, but somewhat different definitions of malaria risk are required for each of the two scenarios just enumerated. In the first case, it is sufficient to define the risk of malaria in terms of the prevalence in the high-transmission area population since the importation is occurring from that population. In the second case, a more sophisticated model is needed that quantifies the risk based on the time a traveller spends in the high-transmission area. Although none of the studies reviewed here used such a model, such models do exist in the literature. In terms of vector-borne diseases, a mathematical model proposed by Massad et al. [50] quantifies the risk of malaria for travellers to areas with stable transmission by considering the duration of exposure and season. The individual risk calculation proposed by Stoddard et al*.* [51] and Tatem et al*.* [52] illustrates the effect of the time spent in risk areas on the chance of dengue and malaria infection, respectively. Moreover, similar time-based models have also been proposed to quantify the risk of exposure to environmental hazards [53, 54].

### Environmental and climatic variables

Environment and climate play an important role in malaria transmission [55–57]. All studies in this review included land use or land cover types that contribute to the distribution of mosquitoes. Various land cover types use used, but forests and villages were the most widely used in the studies. Forests or areas dominated by trees, including crop fields or agricultural plantations, are associated with enhanced malaria transmission because of the appropriate temperature, humidity, and breeding sites for the mosquitoes [58–60], whereas villages and urban areas are associated with lower malaria transmission [28]. For forest areas, detailed characteristics, such as the area of the canopy coverage and the height of the trees, are also used [61, 62].

Satellite imagery has long been used in malaria transmission studies [58, 63–65] and provides a variety of spatial and temporal resolutions [66, 67] without additional cost. However, utilizing the data involves several steps to extract, manipulate, and summarize data and to compute environmental indices [68], which requires expertise from epidemiology and geographic information systems [66]. Approximately 30% of the reviewed studies used satellite imagery to collect data, while the others obtained data from relevant local government agencies. Although data from both sources are acceptable, there is a need to establish a standardized taxonomy of environmental data in the studies. Consider the land-cover type forest as an example. Broadly, it is considered an area without dwellings [29]. At the same time, it can also be characterized in fine-detailed levels as a young, thick, or fallow forest [27]. The differences in the definitions of environment data limit the possibility of repeatability and reusability of the findings from studies.

In addition to land cover, other proxies commonly used to determine malaria transmission include the slope, the altitude, the distance from the breeding sites of mosquitoes (water sources such as a river, paddy field, or forest), and a group of vegetation indices. A moderate slope (less than 12 degrees) [69] is known to facilitate the formation of small running streams or ponds that are appropriate for mosquitoes to breed in [70]. Approximately 8% of studies reviewed included slope in predicting malaria risk. The distance from households or villages to high-risk land cover types such as forests was considered a risk factor for malaria infections in 16% of the reviewed studies. Likewise, evidence shows that villages or households found within a range of mosquito breeding sites or flight ranges (for example, 1.5 km for *An. dirus *[71, 72]) are prone to be high-transmission areas [73, 74], and the use of such distance measurement was observed in 16% of the reviewed studies. The vegetation index, which indicates the vegetation state in a study area, has long been recognized as relevant to malaria transmission [75–77]. Among several available vegetation indices [78], NDVI and EVI were widely used in the spatial modelling of malaria risk [79, 80] and occurred in 8% of the reviewed studies.

Nearly 26% of the reviewed studies directly included climatic factors such as precipitation, humidity, and temperature in estimating malaria risk. In addition, the effect of climatic factors is often indirectly incorporated into the estimation by means of seasonality over the data collection interval [33, 36]. The development of mosquitoes from the aquatic to the adult stage is highly correlated with rainfall and temperature [56, 81, 82]. The studies in this review employed different temporal resolutions of the rainfall and temperature ranging from hourly to annually. Because emerging from pupae to adult mosquito takes approximately 10–14 days, weekly or monthly weather reports are commonly used [81, 83–85]. In addition to disease risk mapping, higher temporal resolutions, such as daily or hourly, are useful in the context of mosquito behaviour, such as the time of night with the highest biting rate [35].

### Human activity and population mobility

Non-environmental factors that are considered to have a pronounced effect on the risk of malaria transmission are human activity and population mobility. In the agricultural sector, both subsistence and commercial farming involve water-harvesting, storage, and irrigation activities that support the breeding of mosquitoes that carry the malaria parasite [86]. Studies that investigated the risks of malaria in rubber plantations [87, 88], paddy fields [86, 89], fruit orchards [90, 91], and palm oil plantations [27, 87] have shown a high prevalence of malaria among the labour force in the agricultural sector. Nearly 30% of the reviewed studies included factors from agricultural settings in their studies.

High population density, urbanization, and poor climatic conditions can force hired hands and workers into swidden farming and logging in forested foothills. Singhanetra-Renard [92] and Dev et al*.* [93] found that workers in swidden farming areas have a high risk of malaria since they are exposed to *Anopheles* mosquitoes that breed in small reservoirs in forested areas and shady clearings on hilly scrub terrain. The taxing physical requirements to commute to the workplace in such terrains have often led to increased logging and subsequent increase in activities such as foraging, fishing, and hunting of seasonal wild produce [94, 95]. Human mobility originating from such high-risk areas poses a continuous risk of malaria introduction into more urbanized and densely populated spaces. Besides activities in agriculture, economic activities in country border areas such as smuggling [92], livestock farming and movement [96, 97], trading of commodities [98, 99], and seeking refuge [100, 101] have been taken into account in determining the malaria risk, and the results show the association with the high rate of malaria infections in populations.

Nearly 30% of the studies included in this review were conducted in border areas, and all of them emphasized the neglected transmission of malaria caused by human mobility. Nonetheless, only one study examined the relationship between mobility and malaria transmission by looking at the relationship between human mobility and the distribution of mosquitoes [30]. Human movement contributes to the circulation of malaria parasites from high-risk areas into areas where local transmission is unsustainable. The calculated risk for non-immune hosts staying longer than 4 months in a high-risk urban setting during peak transmission is only about 0.5% per visit [50]; however, non-immunes who carried out activities in or across the high-risk forest and border areas have been the subjects of large-scale seasonal outbreaks [92, 102, 103]. Imported infections are often the reason for frequent malaria clusters along international borders of Southeast Asian countries, as most of these countries share long land borders with a typical topography consisting of mountain ranges and rivers [104].

Failure to consider population movement contributed to the failure of malaria eradication campaigns in the 1950s and 1960s [105]. Similarly, cross-border malaria hinders countries from achieving malaria elimination [106]. For the latter, consider Thailand as an example. Although most of Thailand is malaria-free, it has yet to achieve malaria elimination since the border region shared with Myanmar continues to have endemic malaria [15, 48, 49]. Due to the diversity of human mobility patterns at different spatial scales [107], acquiring mobility data is a challenging task. Quantification of human mobility has been carried out through epidemiological surveillance data [108], parasite genetic data [109], self-reported travel surveys [99], interviews [108, 110], GPS trackers [111], and anonymized mobile phone data [112]. Surveys and interviews are the principal methods for identifying imported cases, but they can be unreliable and limited due to the scope of memory bias [113]. On the other hand, tracking personalized positions to high temporal and spatial resolution with mobile GPS data is non-trivial. In fact, malaria risk may increase as a result of a combination of different forms of mobility, as well as other factors unrelated to population movements [114, 115].

### Statistical models

In this review, 70% of the studies used types of generalized linear models (GLM), which are designed to generalize linear regression models to investigate non-linear relationships between dependent and independent variables [116]. GLMs also accept a variety of distributions that describe the dependent variables, including Poisson, binomial, and normal, using link functions. Dependent variables in GLMs can be of two types: continuous and discrete. GLMs are easily interpretable and considered flexible as they facilitate the addition of proxies such as socioeconomic factors [117], human mobility indicators [48], seasonality [50], and the use of prevention methods [118] to predict malaria transmission. As the predictors can be incorporated easily, GLM models are prone to include highly correlated independent variables in the models, such as NDVI and rainfall [119, 120] or NDVI and land surface temperature [121, 122]. The presence of multicollinearity between independent variables can lead to an inaccurate estimation of the relationship between the independent and dependent variables [123, 124]. Crucially, predictors must be examined for collinearity, and six studies performed such a test in the variable selection process [15, 18, 23, 26–28]. It is also important to note that when an independent variable that changes over time is included, GLMs are known to be sensitive to autocorrelation in errors [125, 126]. Although it is essential to explore the effect of autocorrelation, only one study in this review conducted the autocorrelation analysis [20].

A variety of spatial resolutions are used to measure the intensity of malaria transmission, including at the provincial [24, 127], regional [21, 128], and village levels [14, 17, 27]. Nearly 50% of studies that used a GLM in this review adopted the highest spatial resolution at the village level to investigate malaria transmission in low-endemic settings. Meanwhile, the rest of the studies that used a GLM utilized a low-temporal resolution for weather (annual) with a low spatial resolution (regional). These studies tended to conduct longitudinal data collection to capture the effect of seasonality on malaria transmission, which is pointed out as a limitation in previous studies [108, 110, 113].

In addition to the GLMs, 9% of the reviewed studies employed approaches that originated from Bayesian statistics. The Bayesian approach estimates the posterior distribution using priors and the observed data described by the likelihood function [129]. The prior distribution in malaria transmission is often determined based on expert opinion [130, 131] or inferred from previous work [30, 132]. Although a weakly informative prior is acceptable [129], an inappropriate prior has an effect on the goodness of fit between the prior distribution and the observed data [133]. There is no standard approach to choosing an appropriate prior, but an alternative is to use the prior predictive p-value [134] or Bayes factor [135] to measure the goodness of fit of the selected prior distribution. The posterior distribution is presented with the mean and its credible interval. The accuracy of the posterior distribution is determined by comparing the similarity between the posterior distribution and the observed data distribution [136] or posterior predictive p-value [137]. Two studies in this review did not utilize such techniques for prior and posterior distributions. One possible reason could be the scarcity of available observed data, such as the biting rate of mosquitos [30] and the prevalence of malaria [10] in low-transmission areas. Like the regression approaches, studies with the Bayesian approach need to exclude the unnecessary independent variables with proper techniques such as a collinearity test [123, 124].

Other approaches to investigate the relationship between environment, weather, and the risk of malaria infection include the use of simple correlation analysis and MCDA [138, 139]. Correlation is widely used to explore the relationship between malaria prevalence and the environment due to its simplicity and ease of interpretation [84, 140, 141]. In addition to serving as the main analysis, correlation can be utilized in data exploration and variable selection. Although MCDA requires the elicitation of expert opinion and evidence from previous work, it has the potential to serve as a guideline when field data is absent.

### Issues in low-transmission areas

In low-transmission areas, asymptomatic malaria infections obstruct achieving zero local malaria transmission. Despite the typically small number of asymptomatic malaria infections, they can cause malaria outbreaks in near-elimination areas [142]. Asymptomatic infections become an issue because the standard approach to reporting malaria infection comes from passive case detection (by microscopy or rapid diagnosis test-RDT), which misses asymptomatic cases [142]. This review shows that the majority of the studies examined use reports from passive cases detection [15–22, 24, 25, 27]. In contrast, active surveillance requires utilizing sophisticated techniques such as molecular screening methods or conducting follow-up longitudinal studies with a relatively large sample of the population [143–145].

In low-transmission settings, two neighboring areas can have different malaria transmission rates [47, 146]. An area with high malaria transmission can be considered a source and its counterpart a sink [102]. Since hotspots can be relatively localized in low transmission areas, data collection should be carried out with high spatial and temporal granularity. This review shows that the highest granularity of data collection on malaria prevalence is at the household level [9–12]. However, most studies investigate the relationship between environmental and meteorological factors and malaria transmission collected at the village level [13–19, 25–29, 31–33]. The environmental and climatic factors are collected either from satellite images or weather stations because these data collection approaches require less manpower and budget than conducting observations in the actual areas of interest [66]. These approaches to data collection are the only solution in some situations where the areas of interest are distant from each other or almost impossible to reach, such as villages in dense forests or villages in neighboring countries [147–149].

## Conclusion

There is no standard definition of the risk of malaria, but most studies in this review adopted the malaria infection rate in humans. Furthermore, malaria transmission highly depends on environmental and climatic factors in several ways, yet neither general guidelines for collecting the environmental and climate variables nor the general definition are shared among the studies. Most reviewed studies utilized GLMs to predict risk based on these factors due to the simplicity and flexibility of the models, yet did not perform the collinearity test before fitting the GLM models. Most of the studies were carried out in either a cross-sectional design or case–control studies, and most utilized OR to report the relationship between exposure to risk and malaria prevalence, which unlike relative risk is not a probability [150, 151] and thus can be difficult to interpret in terms of risk.

In near-elimination settings such as Southeast Asia, malaria proceeds to decline, but the region has encountered a number of challenges to its elimination. One challenge is the detection of asymptomatic infections, which is infeasible on a population scale due to the lack of resources. Routine monitoring of malaria infections over a long period in border areas can also be tedious due to the high level of cross-border mobility, which is difficult to monitor in Southeast Asia because of the large border areas without tight control. Accurately identifying hotspots of malaria infection is also extremely crucial. When combined with human mobility, sources of infection can be revealed. However, regular observation is challenging in border areas, for example, when a destination is deep in forests or outside a country. An important component in quantifying risk is an estimate of the population density of *Anopheles* mosquitoes. However, current approaches, such as larval counts and the use of light traps, are too labour-intensive to use on a routine, widespread basis. These challenges imply the necessity for new approaches to monitoring, prediction, and response to provide more rapidly actionable information to guide national malaria control programmes.

### Recommendations

Following from the observations above, a number of recommendations are derived as guidelines for future studies.

- A more standardized definition of malaria risk would help in comparing and sharing results.
- Given the lack of standards, an explicit description of environmental and climatic variables used in a study could serve as a guideline for further studies.
- The collinearity test should be performed before fitting the GLM models since minimizing the existence of collinearity in the models improves the results and their interpretation.
- Unlike the Relative Risk (RR), Odds Ratio (OR) is not a probability and thus both the OR and RR should be provided in reporting results.
- Research and development are needed into new approaches to monitoring and prediction, such has integration of human mobility in malaria prediction [52, 152], mosquito monitoring using acoustic sensors [153] or images [154], and novel prediction models [149, 155].

This review has described the definition of risk and explored the characteristics of environmental and climatic factors used for its prediction in studies in Southeast Asia. Many of the findings are applicable to other low-transmission settings and could serve as a guideline for further studies of malaria in other regions.

## Data Availability

Data sharing is not applicable to this article as no datasets were generated or analysed during the current study.
